# Low temperature upregulating HSP70 expression to mitigate the paclitaxel-induced damages in NHEK cell

**DOI:** 10.7717/peerj.14630

**Published:** 2023-01-17

**Authors:** Liang Chen, Yi Xu

**Affiliations:** 1 Institute of Biothermal Science & Technology, University of Shanghai for Science and Technology, Shanghai, China; 2 Shanghai Co-innovation Center for Energy Therapy of Tumors, Shanghai, China; 3 Shanghai Technical Service Platform for Cryopreservation of Biological Resources, Shanghai, China

**Keywords:** Scalp cooling, Optimal cooling temperature, Differentially expressed genes, HSP70

## Abstract

Scalp cooling is the most approved treatment for preventing chemotherapy-induced alopecia (CIA). However, the protective mechanism of scalp cooling has rarely been reported. The goal of the present study was to study the relationship between paclitaxel concentration and temperature and the inhibitory effect of low temperature on paclitaxel-induced alopecia. The results showed that the dose of paclitaxel should not exceed 60–70 mg/mL during scalp cooling treatment, and the optimal cooling temperature under different paclitaxel concentrations was determined. Normal human epidermal keratinocytes (NHEK) cells were analyzed by global transcriptome analysis, functional annotation and pathway analysis of differentially expressed genes (DEGs) and ELISA kit to analyze the mechanism of low temperature therapy. The expression of HSPA8, HSPA1A and HSPA1B, which belongs to HSP70, was up-regulated by low temperature. These genes are important target genes of low temperature treatment, which were confirmed by ELISA. The up-regulation of PLK2 and the down-regulation of TXNIP expression are the upstream of mitochondrial dysfunction and ROS, inhibiting the accumulation of ROS and up-regulating the mitochondrial membrane potential. Our research partially elucidates the therapeutic mechanism of scalp cooling, which provides a new idea on the drug research and development in CIA.

## Introduction

Paclitaxel-induced alopecia is a common side effect of chemotherapy and one of the most unbearable side effects (Belum et al., 2016; Chon et al., 2012; Su et al., 2019). Scalp cooling is the only FDA approved treatment for chemotherapy-induced alopecia (CIA) (Bajpai et al., 2020; Sundar et al., 2020). It has been proven that scalp cooling is effective in preventing CIA, but the effective rate (corresponding to hair loss ≤ 50%) of scalp cooling is only about 50% (Kruse & Abraham, 2018; Ross & Fischer-Cartlidge, 2017). A deep understanding of the protective mechanisms of scalp cooling is helpful to improve the effects of treatment.

There is no unified standard for the temperature selection of scalp cooling. The results of Gianotti et al. (2019) showed that the scalp temperature of 18 °C could prevent CIA with 50%∼70% success rate. The temperature of the Paxman Scalp Cooling System used by Smetanay et al. (2019) was 3∼5 °C. Scalp cooling from 30 min before chemotherapy to 90 min after chemotherapy preserved hair in 39.3% of patients. The drug concentration of clinical chemotherapy is different due to the different physiological states of patients, which was not considered in previous studies. For different chemotherapy regimens, there should be different treatment temperatures to determine the relationship between paclitaxel concentration and scalp temperature for achieve accurate treatment. Recently, we have found that cooling significantly rescued M-HFK cells, and that the lower the temperature, the better the protection. Cooling balanced the effects of paclitaxel on cell cycle, restored the cell cycle to normal and induced the up-regulation of HSP70 expression, which had a significant protective effect on cells (Chen, Xu & Ye, 2022).

It is well known that the concentration of HSP70 will increase when the body is stimulated by temperature (Maikova et al., 2016). Franklin et al. (2005) found that HSP70 was highly induced in glial cells and neurons when subjected to harmful stimuli, which played an anti-apoptosis and chaperone role and effectively protected cells of the central nervous system. Hendriks et al. (2019) believed that reducing the temperature will reduce the production of renal ROS, which was helpful to improve the effect of renal protection *in vitro* and facilitate subsequent transplantation. Xiao et al. tested the level of HSP70 in lymphocytes of workers exposed to waste gas in a coke oven. The results showed that individuals with high HSP70 showed lower genotoxic damage than others. HSP70 could protect DNA from genotoxic damage caused by coke oven emissions (Xiao et al., 2002). There have been many studies on the effects of hypothermia on the body, but the specific mechanism of low temperature is still unclear. RNA-sequencing (RNA-seq) is an effective method to study the screening of low temperature tolerance genes in animals and plants (Dunning et al., 2013; Guo et al., 2020; Vatanparast & Park, 2021; Zhang et al., 2020). Scalp cooling is also a type of temperature stimulation and the mechanism has not been reported and still needs further investigation.

The purpose of this study is to determine the relationship between paclitaxel concentration and scalp temperature, and to clarify the inhibitory mechanism of low temperature on paclitaxel-induced alopecia. Therefore, the optimal treatment temperature matched with paclitaxel concentration is determined by theoretical calculation. NHEK cells treated with 22 °C hypothermia were used for total transcriptome sequencing to evaluate the effect of cryotherapy by differential expression analysis and functional annotation. In addition, cell-based experiments were conducted to verify the key therapeutic effects of low temperature.

## Materials & Methods

### Cell culture and experimental design

Normal human epidermal keratinocytes (NHEK) were purchased from Shanghai Guandao Biological Engineering Co. Ltd (Shanghai, China). For all the experiments, NHEKs were used at passages 1∼10 to ensure maximum proliferation capacity (Kleszczyński, Zillikens & Fischer, 2016). The NHEKs were cultured in a DMEM culture containing 10% fetal bovine serum. The control group was cultured at 37 °C and the low temperature group was cultured at 22 °C for 2 h (McCarthy et al., 2013). The set temperature of cell incubator is changed to 22 °C. Taxol powder (Shanghai Aladdin Biological Technology Shanghai City, China) was dissolved in 1:1 castor oil and ethanol to make 6 µg/ml mother liquor was stored at −20 °C, and paclitaxel mother liquor was diluted with stroke-physiological saline solution. NHEK cells were treated with paclitaxel for 2 h.

### RNA extraction

A TRIzol kit (Invitrogen, Waltham, MA, USA) was used to purify the RNA from NHEK cells (Amirouche et al., 2021). one mL TRIzol was added to the six well plates with 5 × 10^6^ cells. The plate was shaken for 5 min. Then 250 µL trichloromethane was added. The plate was allowed to stand for 15 min and centrifuged for 15 min at 4 °C. The supernatant was removed and 500 µL of Isopropyl alcohol was added. Handling the other plate with the same procedure as above.

### MRNA library preparation and sequencing

Genomic DNA was removed by using DNase I (Takara, Shiga, Japan), and RNA quality was determined by 2100 Bioanalyser. mRNA enriched with poly-A tail was first enriched from 1ug of total RNA by oligo (dT) beads and then fragmented with fragmentation buffer. Finally, double stranded cDNA was synthesized using Super Script double-stranded cDNA synthesis Kit (Invitrogen, Waltham, MA, USA) with random hexamer primers. After quantified by TBS380, the double terminal RNA-seq sequencing library was sequenced by using Illumina HiSeq xten sequencer (Zhou et al., 2013), which was conducted by Meiji Biological Co., Ltd (Toyko, Japan).

### RNA-seq data analysis

FastQC (v0.11.9) was used for quality control of sequencing data. Adapters and reads with an average quality score of less than 20 after trimming were discarded by Trimmomatic (v0.39). Feature Counts (v1.6.5) was used for gene quantification (Zimmerli et al., 2020). The RNA-seq data described here are accessible *via* BioProject accession numbers PRJNA872966 (https://www.ncbi.nlm.nih.gov/bioproject/872966). The statistical power of this experimental design was calculated in cluster Profiler Power is *q* value ≤ 0.05.

### Differential gene expression and functional annotation analysis

DEGs between two different samples were identified by calculating the expression level of each transcript according to the transcripts per million reads (TPM) method. The differential expression analysis is to use the *Q* value ≤ 0.05, — log2fc —>1 as an expression gene that is considered to be significantly different. The Kyoto Encyclopedia of genes and genomes (KEGG) database was used to enrich the biological functions of DEGs. GO functional enrichment and KEGG pathway analysis were performed by goatools (https://github.com/tanghaibao/Goatools) and kobas (http://kobas.cbi.pku.edu.cn/home.do). *P*-value ≤ 0.05 and FDR ≤ 0.05 was considered as statistically significant (Lanceta et al., 2021).

### Reactive oxygen species (ROS) measurement

The oxidative fluorescent dye dihydroethidium (20 µM DHE; Sigma-Aldrich, St. Louis, USA) was used to assess ROS production in the NHEK cells according to the manufacturer’s instructions. The 10^4^ cells were planted in 96 well plates overnight, paclitaxel solution was added, and incubated at 37 °C. A DHE working solution was added and incubated for 20 min. The plate was washed three times with a serum-free cell culture solution. The fluorescence spectrophotometer (Biotek Synergy H1; Agilent, Santa Clara, CA, USA) was used to detect fluorescence intensity with 488 nm excitation wavelength and 525 nm emission wavelength.

### Measurement of mitochondrial membrane potential (MMP)

The mitochondrial membrane potential detection kit (Solarbio, Beijing, China) uses JC-1 as a fluorescent probe to quickly and sensitively detect the changes of MMP. The cells were planked in 96 well plates overnight, paclitaxel solution was added, and incubated at 37 °C. 100 µL JC-1 working solution was added after cleaning. This was followed by incubation for 20 min at 37 °C. The plate was washed twice with JC-1 dye buffer, and 100 µL culture medium was added. The fluorescence spectrophotometer was used to detect fluorescence intensity with 490 nm excitation wavelength and 530 nm emission wavelength.

### Enzyme-linked immunosorbent assay (ELISA)

The HSP70 ELISA kit was purchased from Shanghai Frankel Industrial Co., Ltd. (Shanghai, China). Each group had 10^4^ cells in a centrifuge tube. The extract was added to the tube. The cells were broken by ultrasound. The tube was centrifuged at 8,000 g 4 °C for 10 min, take the supernatant. 40 µL diluent and 10 µl supernatant were added into the enzyme labeled coating plate, following 30 min of incubation at 37 °C. The plate was washed five times. This was followed by incubation for 30 min at 37 °C using enzyme labeled reagent. Following the same washing procedure as above, the color developing solutions A and B were applied for 10 min. The absorbance at 450 nm was measured following the application of the stopping solution.

## Results

### Cell survival under the synergistic effect of concentrations and cooling temperatures

In order to determine the activity, the results of the cell activity experiment are necessary, the relationship between the activity and the concentration of the drug (Ribba et al., 2005):

\begin{eqnarray*}S=a+ \frac{1}{b+{k}_{d}C} . \end{eqnarray*}

**Figure 1:**
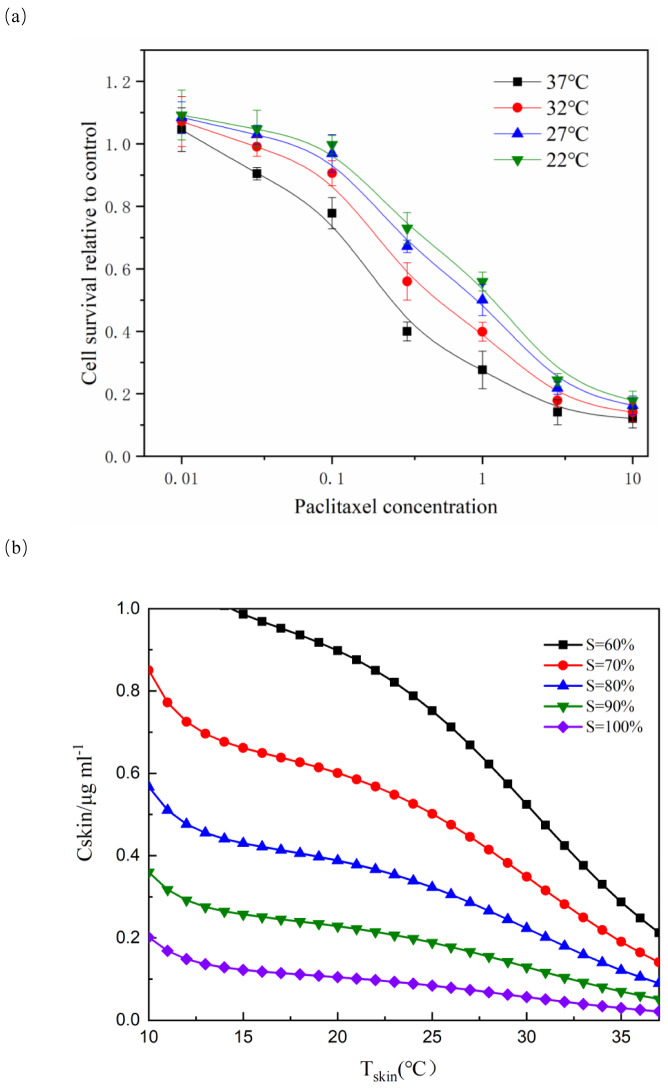
Effect of paclitaxel concentration and temperature on cell survival. (A) After paclitaxel treatment for 2 h, CCK-8 was used to measure cell survival rate. There were three biological replicates for each group. The lower the paclitaxel concentration and the lower the temperature, the higher the cell survival rate (*n* = 3). Data are the mean ± s.e.m. One-way analysis of variance (ANOVA). (B) NHEK cells were treated with different concentrations of paclitaxel and different temperatures. The iso-activity line ranges from a high concentration of taxol at low temperatures to a low concentration of taxol at high temperatures. As the temperature increases, the iso-activity curve becomes S-shaped.

For each temperature group, the parameters a, b and k_d_ were fitted respectively. For each parameter, the function related to temperature is assumed and fitted (Ribba et al., 2005). The fitting result was shown in Fig. 1A. Although only four temperature data points were used, the fitting was meaningful. Due to the limited number of data points, the fitting results must be used carefully.

Figure 1A showed that cell survival was dependent on the concentration of paclitaxel. The higher the concentration of paclitaxel, the lower the cell survival rate. The lower the temperature, the more obvious the protective effect on cells. These results showed that low temperature had a significant protective effect on cells treated with paclitaxel. As shown in Fig. 1B, the isoactivity lines ranged from high concentration at low temperature to low concentration at higher temperature. With the temperature increased, the isoactivity curve showed an S-shaped curve. Caution must be exercised in interpreting the results outside the temperature range of the graph (10 °C < T <  37 °C). It had not been verified experimentally at a temperature below 10 °C. It was expected that the isoactivity line would increase when the temperature was lower than 10 °C.

### Analysis of differential gene expression

There were three high-quality total transcriptome sequencing samples in each group tested in this study. HCA analysis of total transcriptome sequencing data showed that samples from different groups were well separated and had good biological repeatability. Principal component analysis (PCA) provided an overall view of gene expression profile analysis and showed significant differences between all groups.

**Figure 2:**
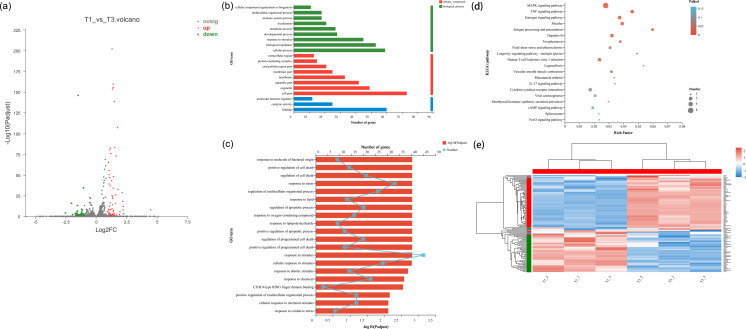
Differentially transcribed genes following low temperature stress treatment of NHEK cells. The same batch of cells were divided into two groups, one was the control group and the other was the 22 °C treatment group. Three biological replicates were used. (A) The DEG volcano plot showed all the sampled genes. Red points represented the up-regulated genes; green points represented the down-regulated genes; grey points represented the not DTGs. (B) Gene ontology (GO) enrichment analysis of DEG. Most consensus sequences were grouped into three major functional categories, namely biological process, cellular component, and molecular function. (C) Go enrichment analysis for DEGs. (D) The KEGG pathway enriched analysis significantly (FDR < 0.05) for the DEGs. Rich factor refers to the ratio of the number of differentially enriched genes to the number of annotated genes in the pathway. The rich factor represents the degree of enrichment. (E) Cluster analysis of DEGs. Red and blue indicated higher and lower expression genes, respectively.

In the cell samples of the control group and low temperature treatment group, a total of 104 DEGs responsed to low temperature treatment were observed, 59 genes were up-regulated and 45 genes were downregulated in NHEK cells (Figs. 2A and 2E). This indicated that the cells responded to low temperature treatment and a certain amount of DEGs. After low temperature treatment, the expression of HSPA8, HSPA1A, HSPA1B, PLK2 and TXNIP changed significantly (Table S1). In addition, we used DEG for GO analysis and KEGG pathway analysis, as well as gene function enrichment.

### GO and KEGG pathway analysis of the DEGs

We performed GO enrichment analysis of DEGs to identify biological processes associated with low temperature. The significant (FDR < 0.05) GO enrichment of DEG in NHEK cells was shown in Figs. 2B–2D. These GO terms included biological process (BP), cellular component (CC) and molecular function (MF) terms.

A total of 41 significant GO terms were obtained, 19 terms were involved in biological processes, 14 and eight terms belonged to cellular components and molecular functions. The main BP included cellular process (*n* = 59), biological regulation (*n* = 53), response to stimulus (*n* = 45), developmental process (*n* = 28) and metabolic process (*n* = 27). The results of GO enrichment analysis showed that under the condition of low temperature treatment, DEGs in the cells were mainly involved in response to stress and stimulus, which was consistent with the situation of low temperature treatment. HSPA8, HSPA1A, HSPA1B, PLK2 and TXNIP all responded to low temperature and participated in response to stimulus Table S2), which was consistent with the genes screened by volcano plot.

KEGG pathway analysis was used to characterize the functional consequences of changes in cell gene expression after low temperature treatment. KEGG pathway analysis showed that DEGs were mainly involved in the regulation of MAPK and TNF signaling pathway with low temperature treatment. HSPA8, HSPA1A and HSPA1B, which were all involved in the MAPK signaling pathway Table S3), proving that HSP70 had an impact on MAPK signaling pathway.

### The effect of low temperature on HSP70, MMP and ROS of NHEK

**Figure 3:**
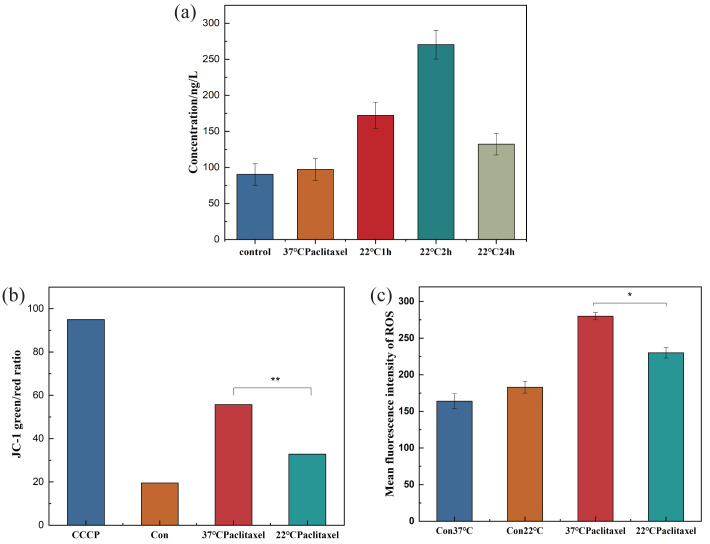
Expression pf HSP70, MMP and ROS of NHEK cells. (A) The concentration of HSP70 was tested using the HSP70 ELISA kit. The concentration of HSP70 increased significantly at 22 °C and firstly increased and then decreased with the increase of exposure time (*n* = 3). (B) The 37 °C and 22 °C groups were all treated with paclitaxel for 2 h. The MMP increased significantly after paclitaxel treatment, the MMP decreased significantly after low temperature treatment (*n* = 3). CCCP group is a positive control group. (C) The ROS of cells significantly decreased after low temperature treatment (*n* = 3). There were three biological replicates for each group. Data are the mean ±s.e.m. One-way analysis of variance (ANOVA). * *P* < 0.05, ^∗∗^*P* < 0.01.

HSP70 has the function of protecting the cells. In order to understand the effect of hypothermia on cellular HSP70, the concentration of HSP70 in the NHEK cells was tested using the HSP70 ELISA kit. The HSP70 level did not change over time in the control group. In Fig. 3A, the concentration of HSP70 was related to the time of low temperature treatment, initially increasing and then decreasing. When the cells were stimulated by low temperature, the HSP70 level in cells increased rapidly and resisted external stimuli. As the stress time lengthens, the cells gradually adapted to the cold, and the expression level of HSP70 decreased. With the extension of stress time, cells would gradually have cold adaptation and the expression level of HSP70 decreased. The concentrations in the control and 2 h groups were 88.351 ng/L and 264.298 ng/L respectively. After low temperature treatment for 2 h, the concentration of HSP70 was about 3 times that of the control group. When cells were stimulated by low temperature, HSP70 in cells increased rapidly, playing a role in resisting external stimuli.

The destruction of MMP could indicate mitochondrial dysfunction, which was the key to the pathway of mitochondrial death induced by chemotherapeutic drugs. To clarify the effect of low temperature on MMP, the MMP of NHEK cells was detected using the ELISA kit. Fig. 3B showed that after paclitaxel chemotherapy, the MMP increased significantly and the mitochondria produced obvious dysfunction. After the low-temperature treatment, the MMP was decreased significantly, but was still higher than that in the control group. The results showed that hypothermia could reduce the MMP and alleviate the mitochondrial dysfunction.

ROS can cause oxidative modification of cellular components such as proteins, nucleic acids and lipids, which can lead to programmed cell death. To prove the effect of low temperature on ROS, the ROS of cells was detected. Low temperature could not be significantly altered in the control group, but paclitaxel chemotherapy resulted in a significant increase in ROS levels. Low temperature treatment significantly decreased the level of ROS, as shown in Fig. 3C.

### The effect of HSP70 inducer and inhibitor on cell survival rate and HSP70 of NHEK

**Figure 4:**
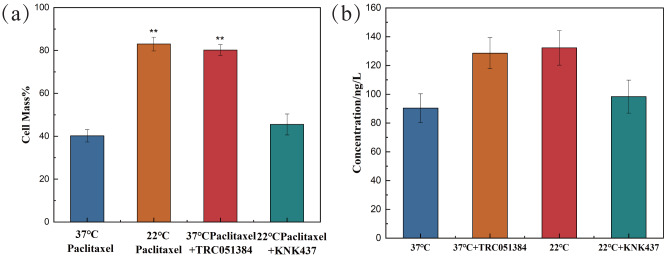
The cell survival rate and expression of HSP70 of NHEK cells. (A) The 10,000 cells were treated with 12.5 µM HSP70 agonist TRC051384 and 100 µM HSP70 inhibitor KNK437 for 2 h (*n* = 3). (B) The 10,000 cells were treated with 12.5 µM HSP70 agonist TRC051384 and 100 *μ*M HSP70 inhibitor KNK437 for 2 h (*n* = 3). The concentration of HSP70 was tested by HSP70 ELISA kit. There were three biological replicates for each group. Data are the mean ± s.e.m. One-way analysis of variance (ANOVA). **P < 0.01.

In the paclitaxel treatment group at 37 °C, the HSP70 agonist TRC051384 was added, and the working concentration was 12.5 µM. The results showed that the cell survival rate was significantly improved, which was basically the same as that of the 22 °C paclitaxel treatment group. The HSP70 inhibitor KNK437 was added in paclitaxel treatment group at 22 °C, and the working concentration was 100 µM. The cell survival rate decreased significantly, reaching the same level as that of the 37 °C paclitaxel treatment group, as shown in Fig. 4A. The above results revealed that HSP70 was the key to the protection of cells by low temperature, but it still needed further verification. The concentration of HSP70 in the cell experimental group was evaluated by HSP70 detection kit. As shown in Fig. 4B, compared with 37 °C group, the concentration of HSP70 in 37 °C and HSP70 inducer group and 22 °C group increased significantly, which proved that both inducer and low temperature could promote the cell synthesis of HSP70. When HSP70 inhibitors were added to the 22 °C group, the concentration of HSP70 was slightly changed compared with that of the 37 °C group, but there was no significant change, which proved that HSP70 inhibitors could significantly inhibit the synthesis of HSP70 in cells. The synthesis of HSP70 was also inhibited even when the cells were under low temperature. HSP70 is a key factor of low temperature therapy.

## Discussion

According to the relationship between cell survival rate and paclitaxel concentration and exposure temperature, the optimum scalp cooling temperature under different concentrations of paclitaxel was determined. The results showed that the survival rate decreased with increasing temperature for a given treatment dose. When the cell survival rate reached 80% or above, we believe that hair loss could be effectively prevented.

**Figure 5:**
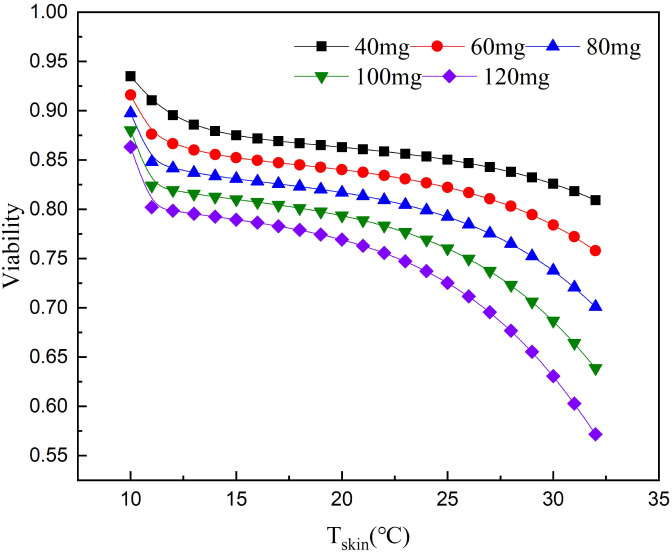
Relationship between scalp temperature and cell survival under different paclitaxel concentrations. The critical treatment temperature under different paclitaxel doses can be calculated.

As seen from Fig. 5, the critical temperature of 40 mg is 32 °C and that of 120 mg is 12 °C. During the scalp cooling process, the average skin temperature is equal to 22 °C. Therefore, the cooling project should be adjusted according to the drug dosage in clinical. However, patients have different tolerance for low temperature, and further cooling is uncomfortable for patients when a certain limit temperature is reached. Therefore, we think that the goal of scalp cooling is that the skin temperature should not be lower than 16 °C. Based on this, and the effective protection of patients’ hair, the dose of the chemotherapeutic drugs should not exceed 60–70 mg.

The DEGs of HSPA8, HSPA1A, HSPA1B, PLK2 and TXNIP were associated with low temperature. HSPA8, HSPA1A and HSPA1B all belong to the heat shock protein family (HSP70). Currently, HSP70 is considered to be the most important HSPs family in mammalian cell HSPs (Mayer & Bukau, 2005; Rosenzweig et al., 2019). HSP70 is a non-specific endogenous protective protein, which can improve cell resistance, inhibit apoptosis and protect against stress (Borges et al., 2012; Mosser et al., 1997; Rérole, Jego & Garrido, 2011). Guihur et al. (2020) found that mild fever can increase the concentration of HSP70 *in vivo*, which prevents apoptosis, protects alveolar cells from inflammatory damage and reduces the mortality of COVID-19 patients. After low temperature exposure, the expression of HSP70 in HUVEC-12 was significantly higher than that in normal cells. The cells were more resistant to apoptosis and necrosis (Guan et al., 2015). The effect of low temperature on the concentration of HSP70 were investigated. Low-temperature treatment increased the concentration of HSP70 in cells, which protected NHEK cells from paclitaxel chemotherapy and reduced the incidence of chemotherapy alopecia. This result is consistent with the clinical results. Low temperature treatment of 22 °C belongs to mild cold stimulus. Cells will adjust themselves to the cold stimulus, so that a certain number of DEGs will be produced. The results of GO enrichment also indicated that DEGs were mainly enriched in cellular process, biological regulation, and response to stimulus. The protective mechanism of HSP70 is to inhibit the expression of stress activated protein kinase, apoptosis gene p53 and Bax, and protect the function of cell mitochondria (Silver & Noble, 2012). HSP70 acts as a molecular chaperone, accelerating the proper folding of peptide chains and protecting cell function. HSP70 can also reduce apoptosis. Therefore, low temperature could reduce the damage of paclitaxel chemotherapy, thus protecting the patient’s hair (Bukau, Weissman & Horwich, 2006; Didelot et al., 2006).

**Figure 6:**
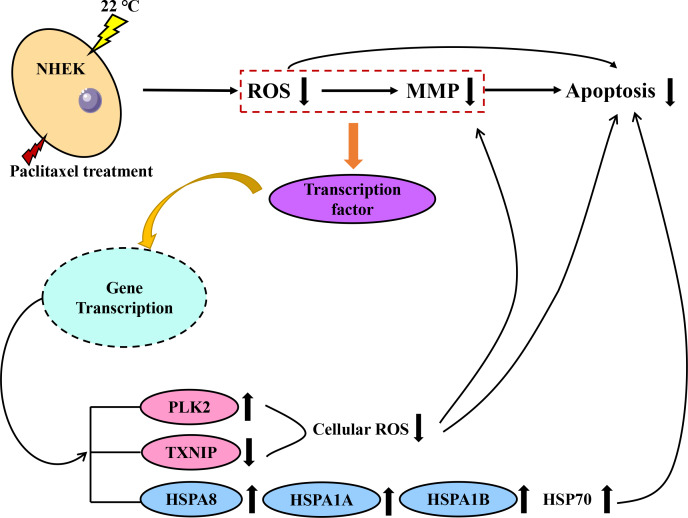
Schematic diagram of paclitaxel damage reduction mechanism by low temperature. When the cells were stimulated by low temperature at 22 °C, the mRNA changed. Red is ROS related mRNA and blue is apoptosis related mRNA.

Both PLK2 and TXNIP are associated with MMP and ROS in cells. The overexpression of PLK2 can promote the growth of many tumor types and inhibit their apoptosis (Zou et al., 2017). The results of Fan et al. (2021) showed that the up regulation of PLK2 had a strong protective effect on retinal ganglion cells (RGC) by weakening the level of ROS. The up-regulation of PLK2 significantly enhanced the activation of Nrf2 signal. The overexpression of PLK2 promotes the phosphorylation of glycogen synthase-3 *β*. Sarina et al. (2019) found that the high expression of TXNIP mediated the increase of ROS and mitochondrial dysfunction. The amount of TXNIP is negatively correlated with the level of ROS. A decrease in TXNIP expression will inhibit oxidative stress and reduce the level of ROS. The effect of hypothermia on ROS and mitochondrial membrane potential of paclitaxel chemotherapy cells was investigated. The results demonstrate that low temperature reduced the level of ROS and the effect on MMP. When cells are treated with paclitaxel, mitochondria are damaged, MMP rises significantly and a large amount of ROS will be released. However, low temperature stabilizes the MMP and reduces the concentration of ROS. The large expression of TXNIP will lead to excessive mitochondrial damage and ROS accumulation. However, after low temperature treatment, the up regulation of PLK2 and the decrease of TXNIP expression inhibit the accumulation of ROS and the up regulation of MMP. In addition, in this study, the addition of the HSP70 agonist TRC051384 can obtain a protective effect similar to that of low temperature, and the protective effect of low temperature on cells disappears after the addition of HSP70 inhibitor KNK437 in the low temperature treatment group. The concentration of HSP70 is also similar to the rule. The results show that HSP70 is the key to protect cells under low temperature. HSP70 agonists can be used as a potential treatment, but further research is still needed. A schematic diagram of paclitaxel damage reduction mechanism by low temperature is shown in Fig. 6. Tissue samples can be used to get more accurate protection mechanisms.

## Conclusion

In conclusion, scalp cooling is the only way to prevent CIA. However, the inhibitory mechanism of scalp cooling is unclear. This study attempts to reveal the inhibitory mechanism of scalp cooling on paclitaxel-induced alopecia. In our work, we found a relationship between paclitaxel concentration, temperature, and cell proliferation to guide the selection of clinical scalp cooling temperature. The results of global transcriptome analysis showed that HSP70 was an important target gene in the process of scalp cooling. Up-regulation of PLK2 and down-regulation of TXNIP inhibited the accumulation of ROS and the up-regulation of MMP. Changes in these genes eventually lead to a reduction in apoptosis, achieving a protective effect on cells and inhibiting alopecia caused by chemotherapy. All the experimental results provide a powerful reference for discovering the protective mechanism of scalp cooling in chemotherapy hair loss. Three HSP70 family genes are key therapeutic targets and drugs can be designed for HSP70 to treat CIA. It is hoped that more in-depth research will be conducted to further reveal the inhibitory mechanism of scalp cooling.

##  Supplemental Information

10.7717/peerj.14630/supp-1 Supplemental Information 1 Differentially expressed genes in cell samples treated at 37° C and 22° C

10.7717/peerj.14630/supp-2 Supplemental Information 2 Differentially expressed genes response to stimulus in GO enrichment analysis

10.7717/peerj.14630/supp-3 Supplemental Information 3 Differentially expressed genes in MAPK signaling pathway in KEGG analysis

10.7717/peerj.14630/supp-4 Supplemental Information 4 Raw figures

10.7717/peerj.14630/supp-5 Supplemental Information 5 Raw data for Figs. 1, 3 and 4

## Additional Information and Declarations

### DNA Deposition

The following information was supplied regarding the deposition of DNA sequences: The RNA-seq data are available at BioProject: PRJNA872966.

### Data Availability

The following information was supplied regarding data availability: The raw data are available in the Supplementary File.
