# Supplementary material for: Low temperature upregulating HSP70 expression to mitigate the paclitaxel-induced damages in NHEK cell
Source: PeerJ. 2023 Jan 17;11:e14630. doi: 10.7717/peerj.14630 (PMC9854382; doi:10.7717/peerj.14630)
Supplement: Supplemental Information 1 [file peerj-11-14630-s001.docx]

Supplementary Table 1 Differentially expressed genes in cell samples treated at 37°C and 22°C.

| Gene_id | Gene name | Log2FC(T3/T1) | Pvalue | Padjust | Significant |
| --- | --- | --- | --- | --- | --- |
| ENSG00000109971 | HSPA8 | 1.306740733 | 4.62E-207 | 6.5E-203 | yes |
| ENSG00000204389 | HSPA1A | 1.383175987 | 2.18E-164 | 1.5E-160 | yes |
| ENSG00000204388 | HSPA1B | 1.379550445 | 6.4E-160 | 3E-156 | yes |
| ENSG00000145632 | PLK2 | 1.325445455 | 6.53E-158 | 2.3E-154 | yes |
| ENSG00000265972 | TXNIP | -1.477421093 | 2.27E-150 | 6.4E-147 | yes |
