# Supplementary material for: Low temperature upregulating HSP70 expression to mitigate the paclitaxel-induced damages in NHEK cell
Source: PeerJ. 2023 Jan 17;11:e14630. doi: 10.7717/peerj.14630 (PMC9854382; doi:10.7717/peerj.14630)
Supplement: Supplemental Information 2 [file peerj-11-14630-s002.docx]

Supplementary Table 2 Differentially expressed genes response to stimulus in GO enrichment analysis.

| Gene id | Gene name | Gene description |
| --- | --- | --- |
| ENSG00000074416 | MGLL | monoglyceride lipase |
| ENSG00000100867 | DHRS2 | dehydrogenase/reductase 2 |
| ENSG00000103546 | SLC6A2 | solute carrier family 6 member 2 |
| ENSG00000104081 | BMF | Bcl2 modifying factor |
| ENSG00000107984 | DKK1 | dickkopf WNT signaling pathway inhibitor 1 |
| ENSG00000109971 | HSPA8 | heat shock protein family A (Hsp70) member 8 |
| ENSG00000115602 | IL1RL1 | interleukin 1 receptor like 1 |
| ENSG00000118503 | TNFAIP3 | TNF alpha induced protein 3 |
| ENSG00000118515 | SGK1 | serum/glucocorticoid regulated kinase 1 |
| ENSG00000118523 | CCN2 | cellular communication network factor 2 |
| ENSG00000120129 | DUSP1 | dual specificity phosphatase 1 |
| ENSG00000122877 | EGR2 | early growth response 2 |
| ENSG00000127863 | TNFRSF19 | TNF receptor superfamily member 19 |
| ENSG00000131016 | AKAP12 | A-kinase anchoring protein 12 |
| ENSG00000134321 | RSAD2 | radical S-adenosyl methionine domain containing 2 |
| Gene id | Gene name | Gene description |
| ENSG00000134326 | CMPK2 | cytidine/uridine monophosphate kinase 2 |
| ENSG00000135114 | OASL | 2'-5'-oligoadenylate synthetase like |
| ENSG00000140465 | CYP1A1 | cytochrome P450 family 1 subfamily A member 1 |
| ENSG00000143878 | RHOB | ras homolog family member B |
| ENSG00000144802 | NFKBIZ | NFKB inhibitor zeta |
| ENSG00000145632 | PLK2 | polo like kinase 2 |
| ENSG00000148677 | ANKRD1 | ankyrin repeat domain 1 |
| ENSG00000148926 | ADM | adrenomedullin |
| ENSG00000162896 | PIGR | polymeric immunoglobulin receptor |
| ENSG00000164400 | CSF2 | colony stimulating factor 2 |
| ENSG00000170345 | FOS | Fos proto-oncogene, AP-1 transcription factor subunit |
| ENSG00000173334 | TRIB1 | tribbles pseudokinase 1 |
| ENSG00000173391 | OLR1 | oxidized low density lipoprotein receptor 1 |
| ENSG00000179388 | EGR3 | early growth response 3 |
| ENSG00000180867 | PDIA3P1 | protein disulfide isomerase family A member 3 pseudogene 1 |
| ENSG00000180871 | CXCR2 | C-X-C motif chemokine receptor 2 |
| ENSG00000184678 | H2BC21 | H2B clustered histone 21 |
| ENSG00000188215 | DCUN1D3 | defective in cullin neddylation 1 domain containing 3 |
| ENSG00000197632 | SERPINB2 | serpin family B member 2 |
| ENSG00000197646 | PDCD1LG2 | programmed cell death 1 ligand 2 |
| ENSG00000204335 | SP5 | Sp5 transcription factor |
| ENSG00000204388 | HSPA1B | heat shock protein family A (Hsp70) member 1B |
| ENSG00000204389 | HSPA1A | heat shock protein family A (Hsp70) member 1A |
| ENSG00000205810 | KLRC3 | killer cell lectin like receptor C3 |
| ENSG00000255150 | EID3 | EP300 interacting inhibitor of differentiation 3 |
| Gene id | Gene name | Gene description |
| ENSG00000264187 | ------ | novel protein |
| ENSG00000265972 | TXNIP | thioredoxin interacting protein |
| ENSG00000275993 | SIK1B | salt inducible kinase 1B (putative) |
| ENSG00000278621 | THBS1-AS1 | THBS1 antisense RNA 1 |
| ENSG00000285106 | ------ | novel transcript |
