# Supplementary material for: Low temperature upregulating HSP70 expression to mitigate the paclitaxel-induced damages in NHEK cell
Source: PeerJ. 2023 Jan 17;11:e14630. doi: 10.7717/peerj.14630 (PMC9854382; doi:10.7717/peerj.14630)
Supplement: Supplemental Information 3 [file peerj-11-14630-s003.docx]

Supplementary Table 3 Differentially expressed genes in MAPK signaling pathway in KEGG analysis.

| gene id | gene name | gene description |
| --- | --- | --- |
| ENSG00000081189 | MEF2C | myocyte enhancer factor 2C |
| ENSG00000092969 | TGFB2 | transforming growth factor beta 2 |
| ENSG00000109971 | HSPA8 | heat shock protein family A (Hsp70) member 8 |
| ENSG00000120129 | DUSP1 | dual specificity phosphatase 1 |
| ENSG00000138675 | FGF5 | fibroblast growth factor 5 |
| ENSG00000170345 | FOS | Fos proto-oncogene, AP-1 transcription factor subunit |
| ENSG00000204388 | HSPA1B | heat shock protein family A (Hsp70) member 1B |
| ENSG00000204389 | HSPA1A | heat shock protein family A (Hsp70) member 1A |
