# Supplementary figures and images for: Low temperature upregulating HSP70 expression to mitigate the paclitaxel-induced damages in NHEK cell
Source: PeerJ. 2023 Jan 17;11:e14630. doi: 10.7717/peerj.14630 (PMC9854382; doi:10.7717/peerj.14630)

## Slide 1
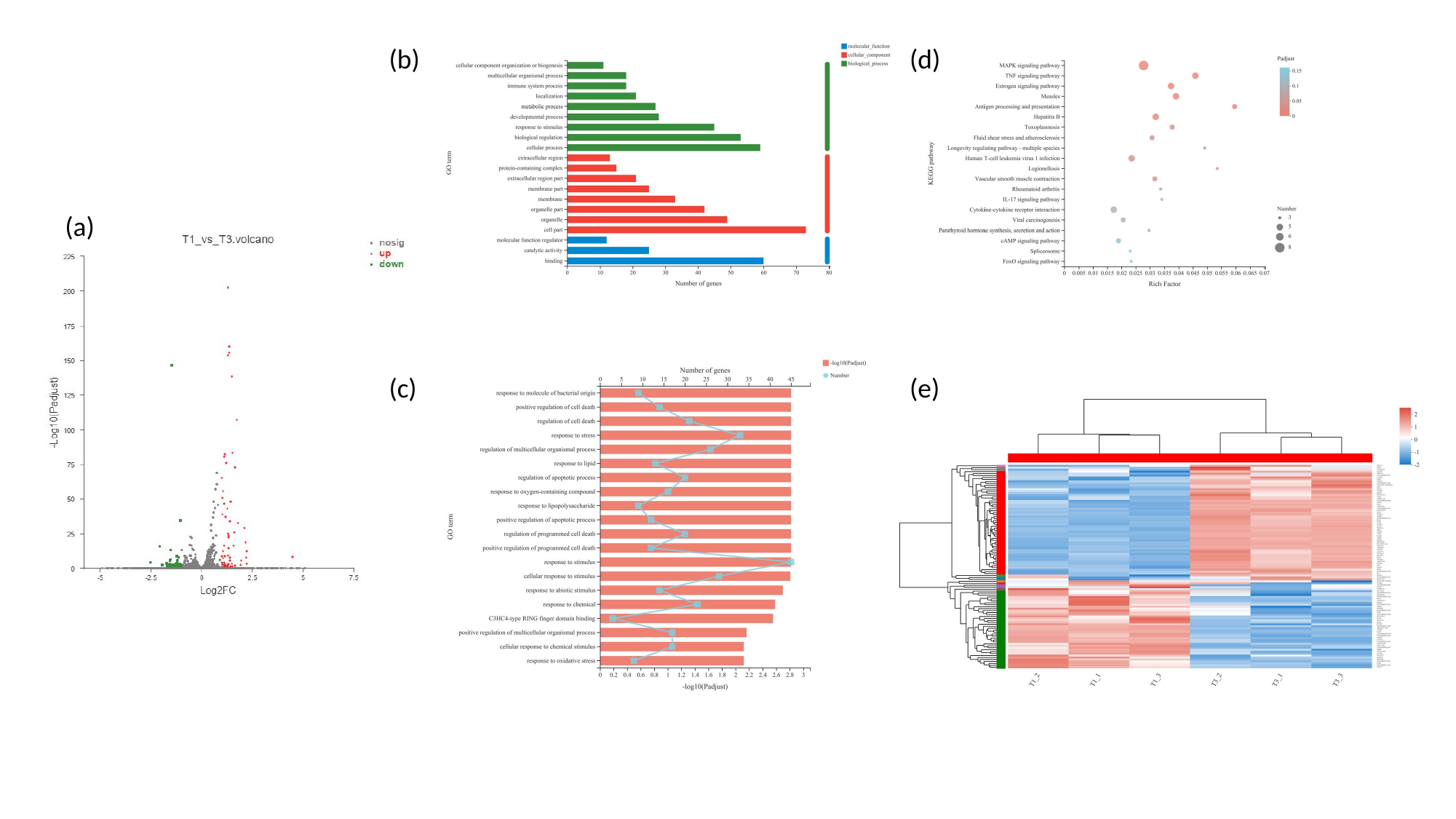

(b)
(d)
(a)
(c)
(e)

Supplement: Supplemental Information 4 [file peerj-11-14630-s004.pptx]
